# An Exploratory Investigation Evaluating the Impact of Fatigue-Induced Stride Length Compensations on Ankle Biomechanics among Skilled Baseball Pitchers

**DOI:** 10.3390/life13040986

**Published:** 2023-04-11

**Authors:** Ryan L. Crotin, Dan K. Ramsey

**Affiliations:** 1 Human Performance Laboratories, Department of Kinesiology, Louisiana Tech University, Ruston, LA 71270, USA; 2 Sports Performance Research Institute New Zealand, Auckland University of Technology, Auckland 1010, New Zealand; 3 Department of Exercise Science, School of Public Health and Health Professions, University at Buffalo, Buffalo, NY 14260, USA; 4 Center for Doctoral Studies and Research, D’Youville University, Buffalo, NY 14260, USA; ramseyd@dyc.edu

**Keywords:** throwing, kinetic chain, compensation, lower body, foot

## Abstract

Altered propulsive and bracing ground reaction forces from lower-body fatigue significantly impact stride length to increase weakness in dynamic elbow stabilizers and risk of medial elbow injury in baseball pitchers. This work investigated altered stride length on three-dimensional ankle joint dynamics to illustrate fatigue-induced changes in ankle motion that can also be impacted by coaching errors. Nineteen pitchers (15 collegiate and 4 high school) were randomized in a crossover design study that encouraged fatigue by throwing two simulated 80-pitch games at ±25% of their desired stride length. An integrated motion-capture system with two force plates and radar gun tracked each throw. Retrospective analysis using pairwise comparisons, including effect size calculations, were undertaken to identify differences in ankle dynamics between stride length conditions for both the drive and stride leg. Longer strides were found to be more effective in drive ankle propulsion and stride-bracing mechanics. Conversely, shorter strides delayed bracing dynamics by demonstrating continued drive ankle plantar flexion moments after stride-foot contact to extend pitchers’ time in propulsion (*p* < 0.001, d > 0.8). Additionally, heightened braking effects were seen during the acceleration phase of throwing with greater stride knee extension power when pitching with shorter strides (*p* < 0.001, d > 0.8). The knowledge gained from this work offers new insight into compensatory stride length adaptation that impacts systemic and throwing arm-specific fatigue to maintain ball velocity, as bilateral ankle joint dynamics can be significantly affected in response to cumulative workload.

## 1. Introduction

Fatigue has been shown to decrease performance in baseball pitchers, where a change in lower-extremity joint power is known to affect throwing arm dynamics that impact the momentum exchange between the throwing arm and trunk, yet can occur with no visible change in ball velocity [1,2,3,4,5,6]. Mechanical changes influenced by fatigue that do not elicit decrements in performance indices (accuracy, ball velocity, FIP, WAR, etc.) may be considered self-organized compensatory adaptations. However, previous studies characterizing biomechanical compensations and pitched ball velocity whilst inferring fatigue and throwing arm injuries should be considered mostly descriptive motor preference investigations as they lack fatigue-inducing protocols. To date, little research on functional baseball-specific fatigue on lower-extremity joint kinematics and kinetics is available. Therefore, specific baseball-pitching fatigue in practice or games should be implemented to accurately and effectively analyze musculoskeletal adaptations that occur during fatigued states to build systematically upon the findings in this specific population.

In studies featuring adolescent athletes, ball velocity was reportedly unaffected by intrasubject differences in normalized stride length, and this relationship was also evident among more proficient pitchers [7,8,9]. Game-based findings suggest collegiate pitchers maintain ball velocities despite stride length variation incurred over cumulative pitches during competition, illustrating the potential of stride length compensation during competition [10]. This was further substantiated where both skilled and unskilled pitchers may alter stride and GRF characteristics to maintain peak ball velocity when faced with physiological stress [11].

Stride length compensation forms an intuitive starting point to evaluate kinetic chain reactions that may precipitate throwing arm injury risks, and stride length variation effects on multiplane and link-segment biomechanics in baseball pitching remains largely unknown. To our knowledge, no study has investigated bilateral 3D ankle joint mechanics in response to altered stride length that may negatively impact kinetic chain interactions and potentially exacerbate throwing arm stress. Therefore, this study is a continuation of a comprehensive full-body biomechanical investigation, with the purpose of retrospectively analyzing how bilateral ankle mechanics are impacted by stride length differences when throwing fastballs during simulated competitive play. The aim was to demonstrate how changes in stride length evoke compensatory ankle biomechanics, stemming from altered propulsive and bracing ground reaction force profiles. For this investigation, it is hypothesized that closed kinetic chain ankle biomechanics would be affected by changes in stride length. Greater ankle joint dynamics (angular displacement, velocities, moments, and joint powers) for both the drive (hind) and stride (lead) ankle are expected to occur earlier in the pitching cycle when adopting longer strides. Drive ankle dorsiflexion is expected to increase with longer strides after stride-foot contact to enact hind ankle braking, as anectodally, toe dragging of the hind foot is common to such deliveries, whereas shorter strides are expected to prolong propulsive drive ankle actions after foot contact and amplify stride-ankle braking dynamics later in the delivery.

## 2. Materials and Methods

Secondary analysis was centered on existing data from the original single cohort, with significant changes in temporal, physiological, ground reaction, momentum, and performance indices having been previously reported [9,11,12,13]. The scope of this investigation focused on ankle dynamics related to stride length that involved skilled competitive baseball pitchers who participated in elite-level high school and collegiate programs.

### 2.1. Participants

Protocols for the subject recruitment, motion capture, and data postprocessing have been previously published [11,12,13,14]. An a priori power analysis was undertaken prior to subject recruitment to suggest that 15 athletes were appropriate for denoting differences in pitching biomechanics due to altered stride length. In brief, 19 healthy and skilled competitive pitchers (aged 18.63 ± 1.67 years, height 1.84 ± 0.054 m, mass 82.14 ± 0.054 kg) from collegiate and high school seasonal travel programs from across western New York participated. Fifteen threw right-handed and four with their left hand. All competed for at least five seasons, were uninjured within two years prior to participation and were medically cleared to participate at time of testing. Each gave informed consent, or parental consent was granted for minors in accordance with the University at Buffalo’s Institutional Review Board (CYIRB DHHS Registration IRB00004088).

### 2.2. Experimental Design

Data collection occurred in the Biomechanics of Human Movement Laboratory at the University at Buffalo and included an integrated biomechanical environment connecting optical tracking with ground reaction force technology in capturing the pitching delivery. Athletes were randomized in being crossed over to throw two simulated 80-pitch games. Subjects delivered the baseball in either an overstride or understride game condition that was set at 25% increased stride length (overstride) and 25% reduced stride (understride) from their desired stride length. Simulated games were held on separate days that were spaced by a 72 h rest window prior to entering a secondary simulated game in the crossover design.

### 2.3. Data Collection

A motion-capture system involving eight cameras (MX20—Vicon Motion Systems, Centennial, CO, USA) synchronized with two floor-embedded force plates (KISTLER Corp., Amherst, NY, USA) were aligned in series so that drive and stride legs contacted opposing force platforms and provided data that tracked the entire pitching delivery complete with fastball and off-speed pitched baseball velocities. Pitch velocities were recorded using a radar gun and LED display (Jugs Sports, Tualatin, OR, USA) that was positioned behind a pitching net. Retroreflective markers defined body segments that were captured at 240 Hz through infrared cameras and 960 Hz for force plate data. An LED screen connected to the radar gun provided visual feedback to encourage athletes to deliver fastball pitches with maximum intent.

After anthropometric measures for height and weight were obtained, retroreflective markers were affixed bilaterally to the whole body, and reflective tape, cut into circles, was secured to the baseball and positioned on the ball in places that were visible to the cameras for the entire arm path that allowed for tracking whole-body motion until the instant of ball release. After static standing and functional hip calibrations, 3D marker trajectories were reconstructed (Vicon Nexus 1.8, Centennial, CO, USA). In total, sixty-three 19 mm and 25 mm retroreflective markers were affixed to body segments for optical 3D tracking, including determining segment inertial properties, subject calibration, and determining hallmark events in the delivery to delineate discrete time points. A hard, sacral thermoplastic shell was tracked optically through three non-collinear markers, which indicated where the pelvis segment was in the 3D space and was positioned over the sacrum using Velcro and elastic overwrap (SuperWrap^TM^, Fabrifoam, Inc., Exton, PA, USA). The elastic wrap was worn securely around the subject’s waist, while ASIS and PSIS markers were attached at the front. The moment of ball release was estimated between the marked ball and throwing hand. For further specifics on anatomical marker placement and subject calibration, please see previous work evaluating stride length impacts on physiological outcomes [11].

### 2.4. Stride Length Determination

A 10 min standard full-body general warmup preceded 3D motion capture, adapted from strength and conditioning programs for professional baseball pitchers in preparation for overhand throwing. Thereafter, each participant threw 30–40 practice balls (Rawlings Group, St. Louis, MO, USA) from flat ground (no pitching mound) into a 6.5′ × 6′ catch net (Rawlings Group, St. Louis, MO, USA) restricted to a distance of 5.69 m owing to lab dimension constraints rather than from the standard 18.4 m. The pitching delivery was standardized to the stretch delivery [15] with throwing intensity progressively increased to 100% effort until the 20th delivery. Motion recordings and ball velocities at the desired stride length were tracked while throwing at 100% effort between the 20th to the 25th delivery. The two highest velocities were averaged, with concomitant desired stride lengths determined from visual inspection of respective kinematic data.

Spatiotemporal parameters were identified from kinematic data and used for desired stride length determination. When the suprapatellar marker of the knee of the stride leg achieved its highest vertical displacement in the windup, it signified peak knee height and was considered the onset of the delivery for kinematic and kinetic analysis. In normalizing bilateral ground reaction forces, body weight was used for both drive and stride leg. Stride-foot contact was determined at the point by which the stride foot landed on the second (opposing) force plate with a vertical ground reaction force that exceeded 5% body weight. Each subject’s desired stride length was calculated as the mean horizontal distance between the drive foot calcaneus at a peak knee height to the stride-foot calcaneus at the stride-foot contact.

Thereafter, desired stride length was adjusted ±25% (determined a priori) to reflect overstride and understride throwing conditions and to challenge throwing mechanics. Absolute stride lengths were expressed in meters as well as normalized percentages across all subjects as a proportion of their body height. In previous work, stride length measures were averaged for overstride and understride conditions and equated to 1.40 ± 0.15 and 0.95 ± 0.14 m, respectively, and normalized as relative measures to body height at 0.76 ± 0.07 and 0.52 ± 0.08% body height. As a result, pitchers required minimal adjustments in their throwing mechanics from their mean desired stride length (1.24 ± 0.17 m and 0.67 ± 0.09% body height) [9]. Statistically significant differences were seen between ±25% stride conditions from the desired stride [12]. Despite stride length differences, the 24% difference in stride length for overstride and understride simulated games fell within 50–80% of the body height (as measured during the warmup) to reflect stride length measurements that are common to collegiate and professional pitchers [3,16,17]. Therefore, comparison of the influence of over- and understride outcomes may be sensitive to genuine throwing conditions.

Force plates embedded in the ground were targeted for subjects to provide constraints for their drive and stride feet so that the desired stride could be visualized and that overstride and understride conditions could be maintained on all throws for each simulated game in the crossover design. For each trial, subjects were acclimated to the respective simulated game stride conditions, and when subjects felt capable of throwing at full effort, the simulated game data collection began. For each recorded pitch, the total delivery time initiated from peak knee height of the lead knee and ended at the point of ball release.

Twenty pitches were thrown per inning—16 thrown at maximum effort for fastballs and 4 off-speed pitches thrown at submaximal effort—that were captured over the course of 4 total innings. Between pitches, subjects were allocated approximately 15 s rest timed by a stopwatch and then provided 9 min of rest between innings. Prior to the start of each inning, five warmup throws were provided, which is consistent with competitive baseball standards. The 80th pitch was the last pitch of the simulated play and terminated the data collection. Means from the two highest fastball velocities in the first and fourth inning that were identified from radar data were analyzed. The rationale for involving both the first and last inning’s highest velocity pitches entailed accounting for potential fatigue impacts over the course of simulated games that could be standardized across all participants as it relates to pitch accumulation.

As previously reported elsewhere [9], ball velocity performance indices remained unchanged over innings accrued (mean fastball velocity 123.5 ± 7.98 km/h vs. 122.7 ± 7.19 km/h and mean change-up velocity 109.1 ± 10.1 km/h vs. 106 ± 9.09 km/h) despite the ±25% stride adjustment, indicating the potential for compensation respective over- and understride conditions.

### 2.5. Data Management and Postprocessing of the Kinematic and Kinetic Data

Following data collection, each trial was analyzed kinematically and kinetically through proprietary software used for advanced biomechanical computations (Visual 3D, C-Motion Inc, Rockville, MD, USA). Calculations involved using custom reduction techniques as previously described [11,13,16]. Briefly, marker trajectories and ground reaction force data were filtered using a second-order bidirectional Butterworth low-pass filter at 13.4 Hz [17,18,19,20] and 40 Hz, respectively. Peak knee height (PKH), stride-foot contact (SFC), maximal external shoulder rotation (MER), and ball release (BR) were identified to delineate hallmark events in the pitching cycle:

- MER: This is the greatest layback of the throwing arm defined from shoulder axial rotation in the kinematic data, and the point when the greatest negative axial humeral rotation occurred to indicate the humerus achieved furthest position into external shoulder rotation.
- BR: Upon visual inspection, ball release occurred coincident with peak linear hand velocity that was inspected visually through both the ball and linear kinematic hand trajectory along the leading axis in the capture environment. As a result, peak linear velocity of the hand indicated ball release and formed the endpoint for the pitching cycle.

With endpoints of PKH and BR determined, the total time per trial was then normalized to 100% of the pitching cycle with PKH corresponding with 0% and BR reflecting the end of the pitching cycle at 100%. Three phases were defined between hallmark events:

(*i*) generation phase: from PKH to SFC
(*ii*) brace-transfer phase: between SFC to MER, and
(*iii*) acceleration phase: within MER to BR

Inverse dynamics resolving 3D ankle joint kinematics and normalized GRF propulsive and bracing data was performed to compute net internal joint moments and powers at four hallmark events (PKH, SFC, MER, BR) and averaged over the generation, brace-transfer, and acceleration phases over the time-normalized pitching cycle. Ankle profiles were based on Newton–Euler equations of motion [21,22,23], with the laboratory frame of reference oriented with the +Y axis directed anteriorly (corresponding to the direction of the intended throw), +Z axis superior, and the +X axis orthogonal to the plane of progression and directed laterally to the right.

### 2.6. Statistical Analysis

Paired *t*-tests for hallmark events and phases were undertaken to evaluate ankle dynamic differences between stride length conditions (overstride vs. understride) using SPSS 19 (SPSS Inc., Chicago, IL, USA). Statistical significance, predetermined a priori, was set at *p* ≤ 0.05 for all statistical tests. To illustrate clinical significance for statistically significant findings, Cohen’s d effect size calculations demonstrated standardized mean differences by denoting trivial (<0.2), small (0.2–0.49), moderate (0.5–0.79), and large (>0.8) effects.

## 3. Results

Triplanar ankle mechanics were found to be significantly different in several pairwise comparisons, with greatest effects seen in the sagittal and frontal plane. Results achieving at least moderate effect size can be seen in accompanying tables (Table 1A,B, Table 2A,B, Table 3A,B), while significant comparisons that met the effect size criteria are discussed in further detail in this section.

**Figure 1:**
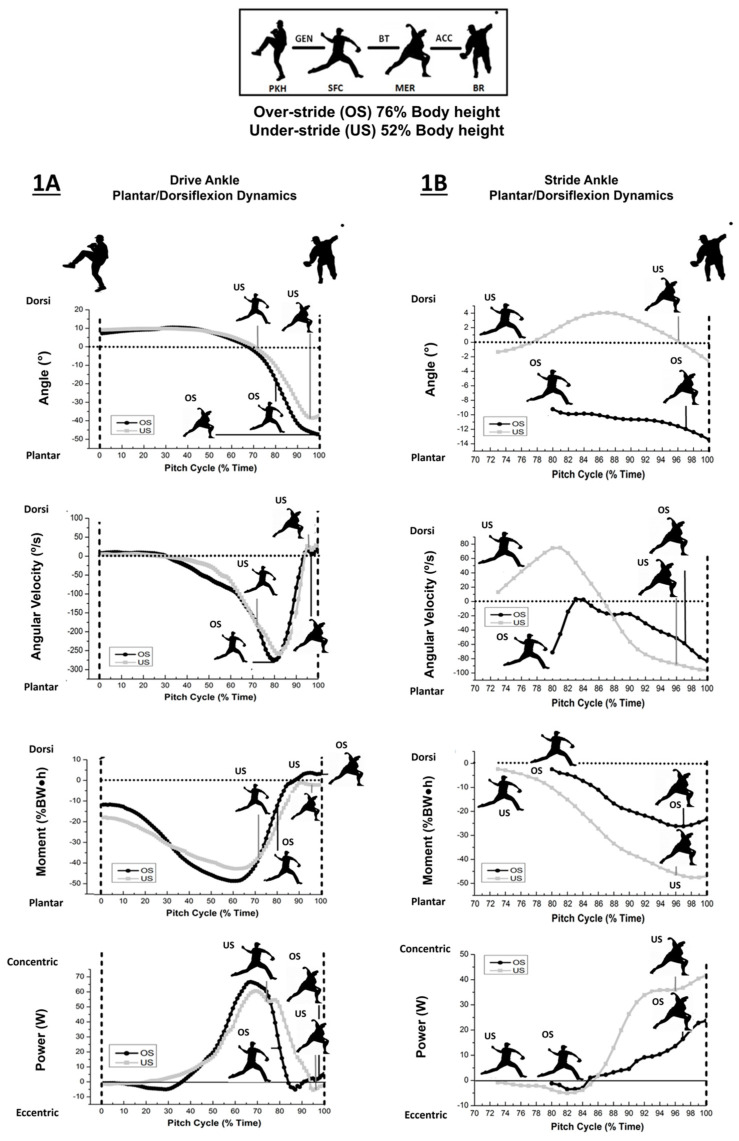
Drive (**A**) and stride (**B**) sagittal ankle plantar flexion/dorsiflexion dynamics.

Ankle dorsi and plantar flexor dynamics for the drive and stride leg are depicted, respectively, in Table 1A,B and Figure 1A,B). The drive (trailing) leg ankle was in greater plantar flexion overall with the longer stride, evident by significant differences at SFC, MER and BR, with greater excursions during brace-transfer and acceleration phases. Longer strides also resulted in significantly higher drive ankle plantar flexor velocities (*p* = 0.015, ES = 0.84) at stride-foot contact and during propulsion (generation phase). Drive leg ankle moments differed at SFC and during the brace-transfer and acceleration phase intervals. Plantar flexor moments were significantly higher (*p* = 0.010, ES = 0.89–1.58) with the shorter strides at SFC and brace transfer, whereas ankle dorsiflexor moments observed with longer strides during the brace-transfer and acceleration phases were statistically different from plantar flexor moments experienced with shorter strides (*p* < 0.001, ES = 1.41–5.45). Longer strides presented significantly higher power generated by the drive ankle’s dorsiflexors during the acceleration phase and at BR (*p* = 0.029, ES = 0.74) whereas shorter stride pitching saw significantly higher power generated by the plantar flexors of the drive ankle during the brace-transfer phase (*p* < 0.001, ES = 1.73) and power absorption during the acceleration phase (*p* < 0.001, ES = 0.66).

As found with the drive ankle, stride leg ankle plantar flexion was statistically higher with a large effect for longer stride lengths throughout the pitching cycle from SFC to BR (Table 1B). In contrast, greater stride ankle dorsiflexion was found during the brace-transfer phase (*p* < 0.001, ES = 6.61) with greater angular plantar flexor velocities observed during the acceleration phase with shorter strides (*p* < 0.001, ES = 3.00). Higher plantar flexor moments throughout the acceleration phase (*p* < 0.001, ES = 20.8) with coincident positive joint powers at BR (*p* = 0.035, ES = 0.71) and during the acceleration phase were experienced with shorter strides (*p* < 0.001, ES = 9.37).

(A) Ankle kinematic conventions described by open kinetic chain (OKC) and closed kinetic chain (CKC) movement indicates plantar flexion (−)/dorsiflexion (+) for the drive ankle. Greater plantar flexion and respective angular velocity were indicated by OS during GEN with concentric dorsiflexion power occurring during ACC. Greater concentric plantar flexion during BT and eccentric plantar flexion during ACC was indicated for US.

(B) Ankle kinematic conventions describing closed kinetic chain (CKC) movement following SFC (73% and 80% time for respective under- and overstride pitching). Stride ankle plantar flexion (−)/dorsiflexion (+); OS indicated greater plantar flexion from SFC to BR. US had greater plantar flexion velocity, moments and joint power during ACC.

**Figure 2:**
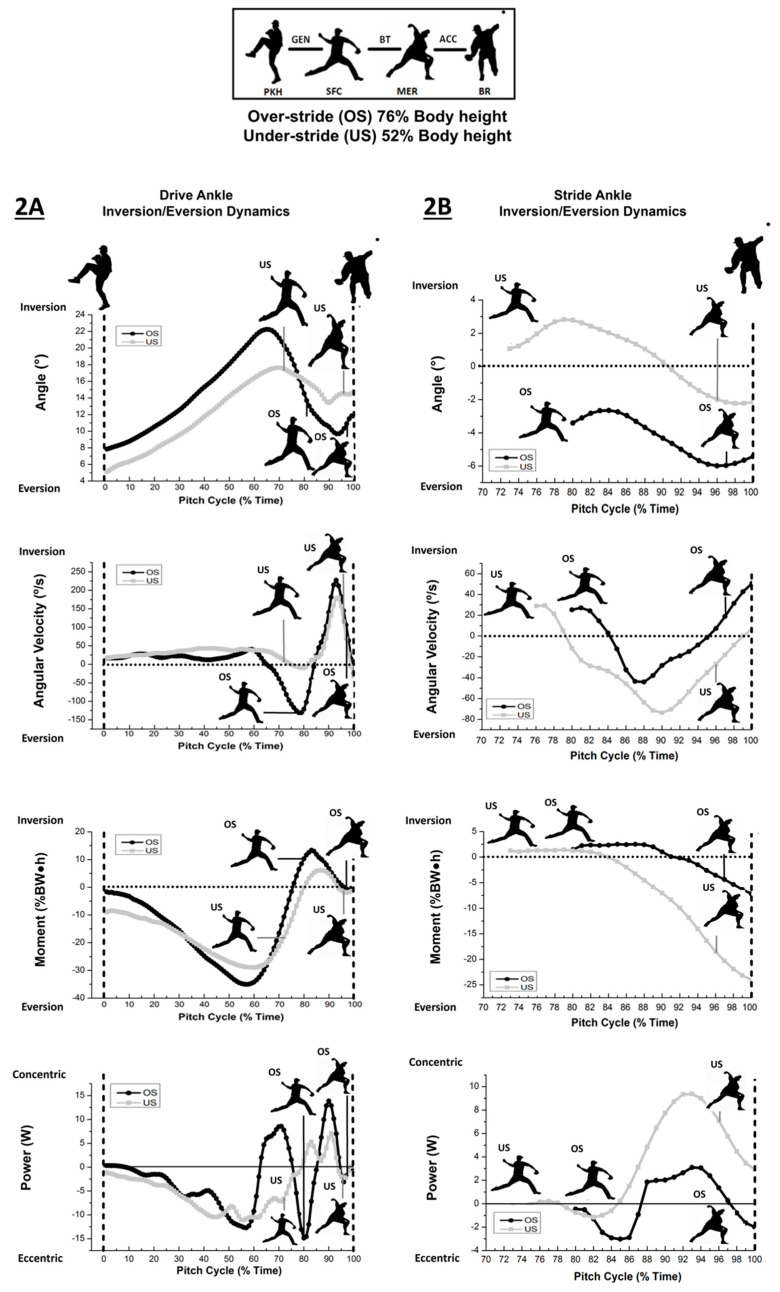
Drive (**A**) and stride (**B**) ankle eversion/inversion dynamics.

Table 2A,B and Figure 2A,B present frontal plane inversion/eversion drive and stride-ankle dynamics with accompanying effect size. Pitching with longer strides resulted in greater inversion of the drive ankle during the generation phase (*p* < 0.001, ES = 0.81), with less inversion (moving to eversion) during BT (*p* < 0.001, BT ES = 3.42) when compared to shorter strides. Inversion angular velocities were shown to be statistically higher for the drive ankle at the generation phase (*p* < 0.001, ES = 0.93) with shorter strides. Internal ankle eversion moments were observed for the drive ankle with shorter strides, as longer strides had shown inversion moments at stride-foot contact (*p* < 0.001, ES = 0.24) (Figure 2A). Longer strides also achieved greater inversion moments during the BT phase (*p* < 0.001, ES = 1.28) (Table 2A).

For the stride ankle, throwing with longer strides demonstrated greater mean eversion during BT and the acceleration phase (*p* < 0.001, BT ES = 2.18, ACC ES = 16.3) compared to shorter strides. Longer stride pitching revealed greater eversion velocities at BR (*p* = 0.034, ES = 0.72) and throughout acceleration the phase (*p* < 0.001, ES = 3.90). After stride-foot contact, mean eversion moments were significantly greater for shorter stride pitching through to BR (Figure 2B) with moderate to large effects (Table 2B). Positive ankle eversion power was evident for increased stride lengths after stride-foot contact with large effect (Table 2B). Illustrated in Figure 2B, positive eversion power was primarily observed during the final 15% of the pitching cycle, although powers were significantly lower for shortened strides during acceleration phase (*p* < 0.001, ES = 2.41).

(A) Ankle kinematic conventions described by open kinetic chain (OKC) and closed kinetic chain (CKC) movement indicates inversion (+)/eversion (−) for the drive ankle. OS indicated greater eversion from SFC to BR and greater inversion velocity during ACC. US indicated a greater eversion moments and joint power from SFC to BR.

(B) Ankle kinematic conventions describing closed kinetic chain (CKC) movement following SFC (73% and 80% time for respective under and overstride pitching). Stride ankle inversion (+)/eversion (−); OS indicated greater eversion from SFC to BR and greater inversion velocity during ACC. US indicated a greater eversion moments and joint power from SFC to BR.

Table 3A,B illustrate internal/external rotation ankle dynamics for the drive and stride ankle. Greater concentric power was generated in external rotation for the drive ankle during the generation phase for longer strides (*p* < 0.001, ES = 0.61). Drive-ankle external rotation velocities were lower during the acceleration phase for longer strides (*p* < 0.001, ES = 2.11). Shorter strides mediated greater mean absorbance (*p* < 0.001, ES = 2.40) with the stride ankle in a more internally rotated position (*p* < 0.001, ES = 9.19) to decelerate the rate of internal rotation during the acceleration phase (Table 3B).

## 4. Discussion

Throwing arm dynamics are influenced by functional strength, range of motion, motor control, and ankle stability in young baseball players [24,25]. Functional ankle instability of the drive ankle (i.e., strength and proprioceptive deficits) concomitant with peak knee height increases postural sway that results in a less stable balance point (center of gravity) during the windup that can alter GRF vectors. Coincident ankle mobility constraints (specifically dorsiflexion range of motion) disrupt center of gravity progression (dynamic balance) and forward momentum generation during the delivery that transfer to the upper extremity, thus predisposing the shoulder and elbow to injury [24,25]. Similarly, the inability to maintain a co-contracted ankle in plantarflexion may reduce braking force magnitude and posterior vector angles that could impact intersegmental force transfer from lead leg bracing to pelvic and trunk rotation.

Given the paucity of evidence connecting ankle pathomechanics to exacerbated shoulder and elbow injury rates in baseball, the present study is the first to analyze bilateral 3D ankle joint dynamics in response to altered stride length. Multiple significant triplanar ankle joint dynamics were evident which confirm the study’s overall and specific hypotheses. Greater ankle joint dynamics were seen earlier in the pitching cycle before stride-foot contact and before the acceleration phase for both the drive and stride ankle, respectively, for longer strides. Similarly, increased drive-ankle dorsiflexion moments for longer strides were evident after stride-foot contact, which is thought to engage hind ankle braking (often resulting in drag lines from the back toe on the pitching mound providing friction), whereas shorter strides prolonged propulsive ankle mechanics after foot contact with abrupt stride-ankle braking observed later in the delivery.

Altered stride length has been shown to affect propulsive and bracing ground reaction force profiles in both magnitude and timing of peaks in a previous study [12], where the results of this work illustrate how both the drive and stride ankle act to regulate respective propulsion and bracing ground reaction forces, momentum transfers and ball velocity, as previously seen [13,16].

Both stride conditions saw maximal drive-ankle plantar flexor moments occurring during 60–65% of the pitching cycle, with peaks close to stride-foot contact observed with the shortened strides as opposed to peaks occurring well before stride-foot contact with the longer strides (Figure 1A). The differences in timing may be responsible for maintaining linear momentum following stride-foot contact, thereby reducing the effect of drive-foot braking for pitchers with shorter strides.

Following stride-foot contact, the drive ankle experienced greater dorsiflexor concentric joint power, since the foot was plantar flexed to a greater extent during the acceleration phase. Increased dorsiflexor power generation with the foot more plantar flexed later in the delivery may be attributed to greater posterior ground reaction forces acting on the drive foot, potentially to increase friction, as previously reported yet not evident with short-stride pitching [12]. The greatest differences observed with shorter strides were during the brace-transfer phase after foot contact, where drive-foot plantar flexor moments owed to power generation in double support (Table 1A). This discrepancy is likely attributable to the amount of time spent in the brace-transfer phase to coordinate propulsive effort and maintain fastball velocity. Previous work indicated that longer strides resulted in less brace-transfer duration (17% time) compared to 23% time with the shorter stride [14]. During the critical instant where throwing arm kinetics are highest, a time point that is better known as the acceleration phase from maximal external rotation to ball release, shorter-stride pitchers saw greater plantar flexor power absorption to resist dorsiflexion, owing to lower posterior ground reaction forces being generated by the hind foot [12]. The plantar flexor moments that persist after stride-foot contact, owing to the abbreviated generation phase, are thought to minimize drive-foot braking and enhance linear and angular momentum through to ball release, as opposed to longer strides, and maintain ball velocity [13,16].

The stride ankle underwent greater dorsiflexion when utilizing the shorter strides, occurring after stride-foot contact and through to maximal external rotation, whereas plantar flexion was maintained throughout the entire pitching cycle with the longer strides and confirmed our secondary hypothesis (Figure 1B). This may be attributed to different landing strategies, evident by forefoot to heel contact and/or heel to flat foot contact when pitching with shorter and longer strides, respectively. Increasing stride-ankle dorsiflexion following stride-foot contact may impact bracing strategies and be identified by changes in magnitude and timing for maximum vertical and posterior ground reaction forces [12]. Shorter strides saw decreased posterior ground reaction forces exerted by the stride foot during the brace-transfer-phase while our results showed increased ankle dorsiflexion, whereas concomitant drive ankle plantar flexor effort increased in promoting propulsion late in the delivery [12]. The result was greater stride-ankle plantar flexor power generation during the acceleration phase through to ball release, which may necessitate more abrupt lead leg braking when pitching with the shorter strides.

Early in the generation phase, drive-ankle inversion was notably higher with the longer strides, whereas inversion was more pronounced and occurred later in the pitching cycle after foot contact when using the shorter strides. When inverted, the drive ankle with longer strides depicted a greater maximal eversion moment (approximately 35% BW•H) (Figure 2A). The greater eversion moments observed during single support are likely attributable to greater propulsive, anteriorly directed ground reaction force and impulse when pitching with longer strides [12]. Compared with shorter strides during propulsive generation, longer stride pitching infers the importance of the force couple between drive-ankle plantar flexion and eversion moments to develop peak anterior ground reaction forces in propulsion before foot contact and appears to coordinate instants single support ankle co-contraction that may precipitate improved energy storage for the knee and hip [12].

Greater stride-ankle eversion angles with the longer stride lengths were seen after stride-foot contact. During this period, eversion kinetics were found to be lower with longer strides (Figure 2B), which is thought to mediate transverse internal pelvic rotation to improve kinematic sequencing. Given greater ankle eversion kinetics and joint powers were noted with shorter strides (Figure 2B), the increased ankle eversion power may induce abrupt bracing to increase pelvic and trunk angular velocities evident during the acceleration phase to maintain ball velocity [13].

In closed kinetic chain lower-extremity movement, internal–external ankle rotation is described by transverse tibial rotations relative to either the planted drive or stride foot. Internal tibial rotation about the planted foot increases external ankle rotation, whereas tibial external rotation denotes internal ankle rotation. The greater contribution of external ankle rotation and respective angular velocities, depicted as greater internal tibial rotation during the generation phase, is thought to augment propulsive effort through the ground reaction free moment. Depending on the direction of the free moment, it acts to resist the tendency of the foot to either abduct (toe out) or adduct (toe in) with respect to the ground. One possible advantage is enhanced fictional force by increasing the ability to apply free moments, which occurs early in the pitching cycle before stride-foot contact. With shortened generation times owing to the shorter stride, the lower external foot rotation moments during propulsion combined with minimal external ankle rotation throughout the pitching delivery may further the need to coordinate drive-ankle propulsive effort following stride-foot contact during double support.

Greater external rotation stride-ankle moments for shorter strides during acceleration (signifying greater internal tibial rotation moments) may be used to distinguish the abrupt deceleration response to slow down closed chain internal ankle rotation (external tibial rotation). The combination of stride-ankle eversion and internal tibial rotation moments during the acceleration phase may be compensatory for the short stride’s reduced transfer of linear energy to rotation, where such lead ankle biomechanics may enhance angular pelvic and trunk momentum to maintain peak ball velocity [13].

Obvious limitations of this work included the following: (1) research was conducted indoors on flat ground, (2) throwing deliveries occurred at shorter distances, (3) pitching trials did not involve a batter or catcher, and (4) pitchers were not wearing cleats. As a result, these limitations prevent generalizability to live game situations. However, despite these limitations, overhead pitching from flat ground is commonly practiced at all baseball levels, substantiating the importance of this study and making it more representative of pitchers’ training regimens.

Among more physically mature pitchers, mound and flat ground throwing deliveries demonstrate similar biomechanical patterns, yet shoulder and elbow loads are augmented with throwing at maximum distance, whereas recent work reports equivalent arm kinetics between conditions [26,27].

In this work, a simple AB crossover design evaluated exploratory relationships related to ankle dynamics that were influenced by altered stride length. The approach allowed each subject to be their own control, which permitted a lower number of subjects to be studied compared to parallel-group studies. One limitation of this research method was not having comparative data for desired stride length data with the ±25% stride conditions. Desired stride length comparisons increase complexity in this research model, and it is recommended to test this intermediate condition in the future with a more involved research approach at several periods (e.g., ABAC or ABCABC designs). The addition of desired stride length into a three-way analysis could also give rise to parallel-group research frameworks (ABC) to account for larger samples.

In summation, longer strides depicted more advantageous triplanar mechanics in propulsion, whereas shorter strides prolonged propulsion and executed higher bracing dynamics later in the delivery, perhaps to amplify transverse trunk momentum to maintain ball velocity [13]. To protect players from injury and improve performance, baseball-specific programs designed by a multidisciplinary team comprised of physicians, athletic trainers, physical therapists, strength coaches, and sport scientists should include routine ankle mobility and strength evaluations that is believed to be necessary for maintaining proper ground reaction force profiles. Finally, biomechanical assessments should support preventative efforts by the multidisciplinary team in optimizing strength length and functional ankle mechanics for baseball pitchers.

## 5. Conclusions

Biomechanical evaluations for baseball pitching should involve examining ankle function dynamically in addition to clinical testing to ensure ground reaction force applications are effective in propulsion and bracing to transfer force from the ground to throwing hand. As a result, this work strongly recommends high-performance teams in baseball to assess stride length critically through 3D optical tracking methods, as compensatory mechanics or ineffective coaching suggestions can be avoided by including ankle motion analysis in their interpretation of efficient throwing mechanics.

## Figures and Tables

**Table 1:** (A) Drive ankle plantar flexion/dorsiflexion dynamics; (B) stride-ankle plantar flexion/dorsiflexion dynamics.

A								
		Peak Knee Height	Generation Phase	Stride-Foot Contact	Brace Transfer	Maximal External Rotation	Acceleration Phase	Ball Release
**Joint Angle (°)**	*OVERSTRIDE*	7.28 (9.12)	5.47 *,† (6.53)	−18.7 (17.9)	−37.0 **,¥ (8.77)	−47.0 **,¥ (5.06)	−47.2 **,¥ (0.19)	−47.1 **,¥ (8.93)
	*UNDERSTRIDE*	9.11 (6.54)	7.73 (3.12)	−4.44 (16.1)	−20.3 (12.1)	−37.6 (10.8)	−37.7 (0.82)	−36.5 (7.91)
**Angular Velocity (°/s)**	*OVERSTRIDE*	9.20 (25.1)	−58.1 (80.6)	−270.0 (140.0)	−120.0 (110.0)	10.6 (116.1)	25.7 (19.7)	56.0 (111.0)
	*UNDERSTRIDE*	4.60 (14.5)	−31.4 (52.0)	−170.6 (96.5)	−172.0 (94.9)	5.23 (165.2)	31.2 (13.8)	51.7 (104.0)
**Joint Moments (%** BW*H)	*OVERSTRIDE*	−11.3 (10.8)	−31.1 (13.6)	−10.9 *,¥ (21.1)	0.23 **,¥ (4.04)	3.21 (4.31)	3.33 **,¥ (0.41)	2.72 (4.53)
	*UNDERSTRIDE*	−17.6 (14.1)	−31.7 (8.67)	−31.6 (25.6)	−12.4 (11.5)	−1.88 (9.31)	−1.75 (0.004)	−1.78 (9.80)
**Joint Power (W/** BW*H)	*OVERSTRIDE*	−0.88 (1.67)	19.2 (26.1)	22.4 (48.3)	1.08 **,¥ (5.20)	2.74 (6.77)	3.74 **,£ (1.29)	2.55 (4.95)
	*UNDERSTRIDE*	−1.42 (2.99)	16.3 (21.2)	47.0 (42.8)	28.3 (21.7)	−0.04 (12.6)	−3.09 (0.67)	−2.64 (8.62)
**B**								
		**Peak Knee Height**	**Generation Phase**	**Stride-Foot Contact**	**Brace Transfer**	**Maximal External Rotation**	**Acceleration Phase**	**Ball Release**
**Joint Angle (°)**	*OVERSTRIDE*	−6.89 (20.3)	−4.04 **,£ (3.21)	−9.58 (10.2)	−10.5 **,¥ (0.64)	−11.9 **,¥ (8.67)	−13.2 **,¥ (0.69)	−14.0 **,¥ (8.93)
	*UNDERSTRIDE*	−2.75 (18.2)	−2.37 (1.40)	−1.66 (8.80)	1.92 (1.72)	0.04 (6.95)	−1.94 (0.56)	−3.43 (7.91)
**Angular Velocity (°/s)**	*OVERSTRIDE*	−15.1 (50.6)	−11.8 **,£ (26.9)	−68.0 (142.0)	−25.5 (18.7)	−64.7 (63.6)	−78.1 **,¥ (6.99)	−82.7 (65.6)
	*UNDERSTRIDE*	8.71 (49.7)	0.76 (9.98)	−1.49 (74.2)	0.63 (59.78)	−92.1 (70.2)	−93.7 (2.27)	−94.0 (55.8)
**Joint Moments (%** BW*H)	*OVERSTRIDE*	−0.40 (1.00)	0.27 (0.70)	−1.05 (7.80)	−16.4 (8.00)	−26.1 *,¥ (23.8)	−23.9 **,¥ (1.55)	−22.1 *,¥ (24.0)
	*UNDERSTRIDE*	−0.40 (1.00)	0.04 (0.62)	−1.37 (0.01)	−24.0 (15.3)	−47.7 (0.25)	−47.0 (0.60)	−46.1 (29.5)
**Joint Power (W/** BW*H)	*OVERSTRIDE*	0.06 (0.32)	−0.93 **,¥ (1.64)	−0.01 (6.77)	4.94 (6.20)	20.2 (23.3)	22.2 **,¥ (1.56)	22.4 (28.9)
	*UNDERSTRIDE*	0.08 (0.27)	−0.13 (0.27)	−0.53 (1.08)	10.5 (16.7)	39.0 (37.3.)	39.8 (2.15)	41.7 (25.4)

(A): Mean (SD) for drive ankle: (−) plantar flexion (degrees), angular velocities (degrees/second), moments (%body weight × height), and ankle power absorption (watts/body weight × height); (+) dorsiflexion (degrees), angular velocities (degrees/second), moments (%body weight × height), and ankle power generation (watts/body weight × height). Closed chain rotations occurred from peak knee height to ball release, which were indicated by posterior (increasing plantar flexion) and anterior (increasing dorsiflexion) rotations of the tibia relative to the planted drive foot. Normalized events and phases; peak knee height; stride-foot contact; maximal external rotation, maximal external rotation; ball release; generation phase; brace-transfer phase; acceleration phase. Significant differences indicated (*p* < 0.001) ** and (*p* < 0.05) *. Effect Size; trivial (<0.2) no symbol; small (0.2–0.49) †; moderate (0.5–0.79) £; Large (≥0.8) ¥. (B): Mean (SD) for drive ankle: (−) plantar flexion (degrees), angular velocities (degrees/second), moments (%body weight x height), and ankle power absorption (watts/body weight × height); (+) dorsiflexion (degrees), angular velocities (degrees/second), moments (%body weight × height), and ankle power generation (watts/body weight × height). Closed chain rotations occurred from stride-foot contact to ball release, which were indicated by posterior (increasing plantar flexion) and anterior (increasing dorsiflexion) rotations of the tibia relative to the planted drive foot. Normalized events and phases; peak knee height; stride-foot contact, smaximal external rotation, maximal external rotation; ball release; generation phase; brace-transfer phase; acceleration phase. Significant differences indicated (*p* < 0.001) ** and (*p* < 0.05) *. moderate (0.5–0.79) £; Large (≥0.8) ¥.

**Table 2:** (A) Drive-ankle inversion/eversion dynamics; (B): stride-ankle inversion/eversion dynamics.

A								
		Peak Knee Height	Generation Phase	Stride-Foot Contact	Brace Transfer	Maximal External Rotation	Acceleration Phase	Ball Release
**Joint Angle (°)**	*OVERSTRIDE*	7.84 (5.31)	15.0 **,¥ (4.73)	14.2 (14.1)	11.1 **,¥ (1.20)	10.7 (11.5)	11.7 **,† (0.18)	11.9 (11.1)
	*UNDERSTRIDE*	5.12 (7.62)	11.4 (4.14)	17.1 (20.3)	15.2 (1.20)	14.9 (16.6)	14.6 (0.23)	14.9 (17.4)
**Angular Velocity (°/s)**	*OVERSTRIDE*	16.6 (27.0)	2.92 **,¥ (43.1)	−87.9 (137.0)	87.7 (103.1)	97.5 (114.2)	3.53 (46.5)	−41.4 (150.5)
	*UNDERSTRIDE*	17.1 (16.5)	31.9 (8.79)	19.4 (80.6)	51.5 (68.4)	92.2 (105.1)	1.43 (48.5)	−52.8 (66.9)
**Joint Moments (%** BW*H)	*OVERSTRIDE*	−1.67 (10.3)	−16.5 (12.6)	10.2 **,† (14.2)	7.14 **,¥ (4.92)	−1.27 (2.39)	−0.96 (0.35)	−1.44 (4.76)
	*UNDERSTRIDE*	−8.99 (9.16)	−19.0 (7.18)	−14.2 (19.4)	0.20 (5.90)	−1.62 (2.19)	−1.45 (0.38)	−0.99 (2.88)
**Joint Power (W/** BW*H)	*OVERSTRIDE*	0.32 (4.44)	−3.33 **,£ (5.78)	−15.1 (24.6)	1.96 (8.65)	−1.27 (4.52)	−0.73 (0.87)	−1.97 (5.11)
	*UNDERSTRIDE*	−1.10 (1.98)	−6.43 (3.24)	0.36 (17.3)	1.56 (3.16)	−2.13 (3.57)	−0.35 (1.13)	0.56 (2.17)
**B**								
		**Peak Knee Height**	**Generation Phase**	**Stride-Foot Contact**	**Brace Transfer**	**Maximal External Rotation**	**Acceleration Phase**	**Ball Release**
**Joint Angle (°)**	*OVERSTRIDE*	1.99 (7.89)	1.20 **,¥ (2.83)	−4.62 *,† (6.62)	−4.16 **,¥ (1.28)	−5.97 (7.81)	−5.52 **,¥ (0.28)	−5.18 (6.96)
	*UNDERSTRIDE*	3.89 (7.71)	3.29 (0.92)	1.97 (6.97)	1.06 (1.55)	−1.76 (6.39)	−2.14 (0.09)	−2.00 (5.43)
**Angular Velocity (°/s)**	*OVERSTRIDE*	1.49 (40.26)	−8.50 (15.4)	39.2 (103.0)	−10.0 (23.3)	27.0 **,¥ (31.4)	44.7 **,¥ (10.1)	54.2 (55.7)
	*UNDERSTRIDE*	−15.12 (66.7)	−8.23 (11.3)	48.0 (119.0)	−27.4 (34.8)	−23.0 (63.4)	−1.84 (11.8)	11.3 (63.6)
**Joint Moments (%** BW*H)	*OVERSTRIDE*	−0.26 (0.23)	0.27 (0.70)	1.97 (8.87)	−0.56 *,£ (2.33)	−5.12 *,¥ (17.2)	−6.71 **,¥ (1.13)	−8.00 *,¥ (17.4)
	*UNDERSTRIDE*	−0.22 (0.32)	0.04 (0.62)	1.08 (1.02)	−3.95 (6.36)	−19.1 (15.1)	−22.7 (1.67)	−24.4 (17.6)
**Joint Power (W/** BW*H)	*OVERSTRIDE*	0.01 (0.12)	−0.34 *,£ (0.96)	−0.30 (8.06)	0.40 *,¥ (2.27)	−0.54 (7.49)	−1.52 **,¥ (0.52)	−1.69 (10.6)
	*UNDERSTRIDE*	−0.03 (0.11)	−0.01 (0.14)	0.05 (1.43)	3.09 (4.12)	6.26 (17.6)	3.86 (1.27)	2.65 (23.9)

(A): Mean (SD) for drive ankle: (−) ankle eversion (degrees), angular velocities (degrees/second), moments (%body weight × height), and ankle power absorption (watts/body weight × height); (+) ankle inversion (degrees), angular velocities (degrees/second), moments (%body weight × height), and ankle power generation (watts/body weight × height). Closed-chain rotations occurred from peak knee height to stride-foot contact, which were indicated by negative frontal plane rotations of the tibia with the planted drive foot respect to the ankle joint (increasing ankle inversion) and positive tibial rotation in the frontal plane (in-creasing ankle eversion). Normalized events and phases; peak knee height; stride-foot contact; maximal external rotation; ball release; generation phase; BT, brace transfer; acceleration phase. Significant differences indicated (*p* < 0.001) **. effect size; trivial (<0.2) no symbol; small (0.2–0.49) †; moderate (0.5–0.79) £; large (≥0.8) ¥. (B): Mean (SD) for stride ankle: (−) ankle eversion (degrees), angular velocities (degrees/second), moments (%body weight × height), and ankle power absorption (watts/body weight × height); (+) ankle inversion (degrees), angular velocities (degrees/second), moments (%body weight × height), and ankle power generation (watts/body weight × height). Closed-chain rotations occurred from stride-foot contact to ball release, which were indicated by negative frontal plane rotations of the tibia with respect to the planted stride foot (increasing ankle inversion) and positive tibial rotation (increasing ankle eversion). Normalized events and phases; peak knee height; stride foot contact; maximal external rotation; ball release; generation phase; BT, brace transfer; acceleration phase. Significant differences indicated (*p* < 0.001) ** and (*p* < 0.05) *. Effect size; trivial (<0.2) no symbol; small (0.2–0.49) †; moderate (0.5–0.79) £; large (≥0.8) ¥.

**Table 3:** (A) Drive-ankle internal/external rotation dynamics; (B): stride-ankle internal/external rotation dynamics.

A								
		Peak Knee Height	Generation Phase	Stride-Foot Contact	Brace Transfer	Maximal External Rotation	Acceleration Phase	Ball Release
**Joint Angle (°)**	*OVERSTRIDE*	14.5 (18.1)	9.76 (6.82)	−11.8 **,† (13.8)	−0.43 (10.6)	15.8 (16.5)	15.6 (0.16)	15.4 (13.7)
	*UNDERSTRIDE*	9.64 (13.4)	11.4 (1.63)	7.11 (11.8)	4.27 (4.54)	14.7 (22.5)	14.9 (1.20)	13.3 (20.8)
**Angular Velocity (°/s)**	*OVERSTRIDE*	−17.1 (73.0)	−31.3 **,† (50.0)	−52.9 (170.0)	166.2 (131.5)	16.2 (248.0)	−21.2 (19.8)	−44.3 (219.3)
	*UNDERSTRIDE*	33.1 (62.9)	−10.8 (44.8)	−119.0 (154.2)	96.7 (170.6)	7.35 (327.3)	−92.2 43.3)	−72.0 (282.6)
**Joint Moments (%** BW*H)	*OVERSTRIDE*	−6.95 (10.7)	−5.00 (9.24)	14.4 (13.5)	4.98 (5.65)	−2.56 (2.89)	−1.94 (0.54)	−1.13 (2.93)
	*UNDERSTRIDE*	−10.4 (6.13)	−7.01 (5.56)	10.1 (12.8)	9.11 (6.37)	−1.38 (3.51)	−1.39 (0.29)	−1.03 (2.96)
**Joint Power (W/** BW*H)	*OVERSTRIDE*	−1.25 (5.31)	−5.69 **,£ (7.13)	−10.8 (32.5)	5.09 (9.03)	−0.11 (5.79)	−0.68 **,¥ (0.42)	−1.27 (2.99)
	*UNDERSTRIDE*	−5.65 (12.6)	−2.37 (2.90)	−12.8 (28.3)	−2.80 (15.5)	1.57 (7.24)	2.70 (1.55)	2.79 (11.6)
**B**								
		**Peak** **Knee** **Height**	**Generation Phase**	**Stride** **Foot Contact**	**Brace Transfer**	**Maximal External Rotation**	**Acceleration Phase**	**Ball Release**
**Joint Angle (°)**	*OVERSTRIDE*	10.9 (23.8)	7.64 (3.20)	4.52 (18.0)	9.38 (3.17)	14.5 (19.9)	15.0 **,¥ (0.65)	15.7 (22.9)
	*UNDERSTRIDE*	7.94 (14.9)	8.27 (3.68)	0.01 (21.6)	8.21 (6.21)	20.4 (22.5)	22.7 (0.99)	23.7 (25.9)
**Angular Velocity (°/s)**	*OVERSTRIDE*	−0.55 (55.2)	−9.57 **,£ (32.3)	26.1 (110.0)	90.0 **,¥ (22.7)	84.6 (167.0)	85.2 (7.67)	74.5 (165.0)
	*UNDERSTRIDE*	−8.02 (48.6)	−26.6 (27.8)	25.2 (112.0)	144.0 (73.3)	177.0 (140.1)	112.0 (39.0)	62.1 (182.0)
**JointMoments (%** BW*H)	*OVERSTRIDE*	−0.20 (0.22)	−0.14 (0.54)	1.20 (3.06)	−0.23 (2.81)	−6.73 (8.08)	−7.76 **,¥ (0.71)	−8.49 (9.16)
	*UNDERSTRIDE*	−0.23 (0.41)	0.06 (0.74)	2.39 (2.27)	1.43 (5.37)	−11.7 (8.14)	−14.1 (1.16)	−15.2 (7.06)
**Joint Power (W/** BW*H)	*OVERSTRIDE*	−0.01 (0.14)	0.20 (0.32)	−1.32 (5.58)	−2.43 (4.05)	−11.8 (19.1)	−13.2 *,¥ (0.71)	−12.5 (23.6)
	*UNDERSTRIDE*	0.03 (0.22)	0.11 (0.19)	1.08 (3.70)	−2.45 (5.37)	−20.2 (23.7)	−18.0 (2.74)	−14.2 (28.5)

(A): Mean (SD) for drive ankle: (−) ankle external rotation/foot abduction (degrees), angular velocities (degrees/second), moments (%body weight × height), and ankle power absorption (watts/body weight × height); (+) ankle internal rotation/foot adduction (degrees), angular velocities (degrees/second), moments (%body weight × height), and ankle power generation (watts/body weight × height). Closed-chain ankle internal rotation occurred from peak knee height to stride-foot contact (planted drive-leg tibia moving with a negative transverse rotation with respect to the drive foot). Normalized events and phases; peak knee height; stride foot contact; maximal external rotation; ball release; generation phase; BT brace transfer; acceleration phase. Significant differences indicated (*p* < 0.001) **. Effect Size; Trivial (<0.2) no symbol; small (0.2–0.49) †; moderate (0.5–0.79) £; large (≥0.8) ¥. (B): Mean (SD) for stride ankle: (−) ankle external rotation/foot abduction (degrees), angular velocities (degrees/second), moments (%body weight × height), and ankle power absorption (watts/body weight × height); (+) ankle internal rotation/foot adduction (degrees), angular velocities (degrees/second), moments (%body weight × height), and ankle power generation (watts/body weight × height). Closed-chain rotations occurred from stride-foot contact to ball release, which were indicated by negative transverse rotations of the stride leg tibia (increasing internal ankle rotation/food adduction for the planted stride foot). Normalized events and phases; peak knee height; stride foot contact; maximal external rotation; ball release; generation phase; BT, brace-transfer; acceleration phase. Significant differences indicated (*p* < 0.001) ** and (*p* < 0.05) *. Effect size; trivial (<0.2) no symbol; moderate (0.5–0.79) £; large (≥0.8) ¥.

## Data Availability

Data for this study is not contained in a publicly archived dataset for this study. The data currently remains with identifiers, and therefore is not available for public consumption.
